# Monitoring the reproduction number of COVID-19 in France: estimates compared from 3 datasets

**DOI:** 10.1093/eurpub/ckac129.280

**Published:** 2022-10-25

**Authors:** C Bonaldi, A Fouillet, C Sommen, D Lévy-Bruhl, J Paireau

**Affiliations:** 1 DATA Science Division, French Public Health Agency, Saint Maurice, France; 2 Infectious Diseases Division, French Public Health Agency, Saint Maurice, France; 3 Mathematical Modelling of Infectious Diseases, Institut Pasteur, Paris, France

## Abstract

**Background:**

The effective reproduction number (Rt) represents the average number of secondary cases generated by an infected person. During an outbreak, near-real-time monitoring of Rt constitutes a key indicator for detecting changes in disease transmission and assessing the effectiveness of interventions. The estimation of Rt usually requires identifying infected cases in the population which is in practice challenging from available data. The purpose of this study was to compare Rt estimates for COVID-19 surveillance in France based on three data sources of different sensitivity and specificity for identifying infected cases.

**Methods:**

By applying a statistical method developed by Cori et al., we estimated Rt using (1) confirmed cases identified from positive virological tests among the tested population (2) suspected cases recorded by a national network of emergency departments (3) hospital admissions for COVID-19 recorded by a national administrative system to manage hospital's organization.

**Results:**

From June 2020 to March 2022, the estimates of Rt in France showed similar temporal trends regardless of the dataset. Estimates based on the daily number of confirmed cases provided an earlier signal that the two other sources, with a lag of 3 and 6 days compared to estimates based on emergency department visits and hospital admissions, respectively.

**Conclusions:**

The COVID-19 experience has proven that monitoring temporal changes in Rt was a key indicator to help public health authorities controlling the outbreak in real time. Having data on infected people in the population to estimate the Rt is not straightforward in practice. As this study has shown, the opportunity of using more readily available data, provided that it is highly correlated with the spread of infection, gives a practical solution for monitoring the COVID-19 epidemic and any epidemic in general.

**Key messages:**

• The effective reproduction number (Rt) is a key parameter to monitor transmission during epidemics but its estimation from available data is often a critical issue.

• Based on COVID-19 experience, data sufficiently correlated with the spread of infection may be appropriate to estimate Rt and monitor its temporal trend.

